# Transfer learning between preclinical models and human tumors identifies a conserved NK cell activation signature in anti-CTLA-4 responsive tumors

**DOI:** 10.1186/s13073-021-00944-5

**Published:** 2021-08-11

**Authors:** Emily F. Davis-Marcisak, Allison A. Fitzgerald, Michael D. Kessler, Ludmila Danilova, Elizabeth M. Jaffee, Neeha Zaidi, Louis M. Weiner, Elana J. Fertig

**Affiliations:** 1 McKusick-Nathans Institute of the Department of Genetic Medicine, Johns Hopkins School of Medicine, Baltimore, MD USA; 2 Department of Oncology, Sidney Kimmel Comprehensive Cancer Center, Johns Hopkins School of Medicine, Baltimore, MD USA; 3 Department of Oncology, Georgetown Lombardi Comprehensive Cancer Center, Georgetown University Medical Center, Washington, DC USA; 4 Department of Applied Mathematics and Statistics, Johns Hopkins University Whiting School of Engineering, Baltimore, MD USA; 5 Department of Biomedical Engineering, Johns Hopkins University School of Medicine, Baltimore, MD USA

## Abstract

**Background:**

Tumor response to therapy is affected by both the cell types and the cell states present in the tumor microenvironment. This is true for many cancer treatments, including immune checkpoint inhibitors (ICIs). While it is well-established that ICIs promote T cell activation, their broader impact on other intratumoral immune cells is unclear; this information is needed to identify new mechanisms of action and improve ICI efficacy. Many preclinical studies have begun using single-cell analysis to delineate therapeutic responses in individual immune cell types within tumors. One major limitation to this approach is that therapeutic mechanisms identified in preclinical models have failed to fully translate to human disease, restraining efforts to improve ICI efficacy in translational research.

**Method:**

We previously developed a computational transfer learning approach called projectR to identify shared biology between independent high-throughput single-cell RNA-sequencing (scRNA-seq) datasets. In the present study, we test this algorithm’s ability to identify conserved and clinically relevant transcriptional changes in complex tumor scRNA-seq data and expand its application to the comparison of scRNA-seq datasets with additional data types such as bulk RNA-seq and mass cytometry.

**Results:**

We found a conserved signature of NK cell activation in anti-CTLA-4 responsive mouse and human tumors. In human metastatic melanoma, we found that the NK cell activation signature associates with longer overall survival and is predictive of anti-CTLA-4 (ipilimumab) response. Additional molecular approaches to confirm the computational findings demonstrated that human NK cells express CTLA-4 and bind anti-CTLA-4 antibodies independent of the antibody binding receptor (FcR) and that similar to T cells, CTLA-4 expression by NK cells is modified by cytokine-mediated and target cell-mediated NK cell activation.

**Conclusions:**

These data demonstrate a novel application of our transfer learning approach, which was able to identify cell state transitions conserved in preclinical models and human tumors. This approach can be adapted to explore many questions in cancer therapeutics, enhance translational research, and enable better understanding and treatment of disease.

**Supplementary Information:**

The online version contains supplementary material available at 10.1186/s13073-021-00944-5.

## Background

Single-cell RNA-sequencing (scRNA-seq) data provide an unprecedented opportunity to unravel the cellular complexity and diversity of immune cell populations in the tumor microenvironment [1]. When used in the context of immunotherapy, scRNA-seq data of tumors can provide a more comprehensive understanding of the molecular and cellular pathways that drive therapeutic response and resistance. While studies often use preclinical mouse models as a convenient and useful tool for studying therapeutic response mechanisms, they are limited in their ability to infer biology relevant to therapeutic responses in humans. To improve the clinical efficacy of immunotherapies such as immune checkpoint inhibitors (ICIs), we need a deeper understanding of the fundamental mechanisms that underlie the anti-tumor activity of ICIs in humans.

Many aspects of the immune system are conserved between mice and humans, but there are significant species-specific differences [2]. These differences may contribute to the frequent failure of therapies that are effective in mouse models from showing similar efficacy in humans [3]. Discrepancies between ICI mechanisms observed in mice and humans may be further complicated by species-specific differences that mask detection of conserved alterations in responding immune cells. A deeper understanding of human and mouse immune responses to immunotherapy could generate new insights into properties that define therapeutic sensitivity. Emerging scRNA-seq studies that have begun to characterize changes in gene expression after ICI treatment [4–6] are ideally suited to begin learning these mechanisms. However, computational tools that identify conserved cell state transitions across species are needed to compensate for species-specific immune system differences in transcriptional data. As scRNA-seq becomes increasingly popular in immuno-oncology, such tools will be essential to validate preclinical findings in terms of both robustness and clinical relevance.

To enable cross-species data integration, we previously developed a computational framework that uses matrix factorization (CoGAPS) and transfer learning (projectR) to integrate transcriptional datasets from different species [7]. This approach has led to the identification of both species-specific and conserved biological processes in the developing retina of mice and humans [8, 9], but it has not yet been applied to cancer therapeutics. To determine if transfer learning can identify conserved and clinically relevant transcriptional alterations within the tumor microenvironment induced by therapy, we applied it to learned cellular patterns from scRNA-seq data of intratumoral immune cells in ICI-treated preclinical models and human patients.

We focused our investigation on the impact of anti-CTLA-4 antibodies because of the numerous cellular mechanisms of action of anti-CTLA-4 antibodies (ipilimumab) found to underlie its efficacy [10, 11]. By blocking the inhibitory T cell receptor CTLA-4, anti-CTLA-4 antibodies enhance T cell effector activity, causing tumor regression [12, 13]. Studies in mice suggest that anti-CTLA-4 efficacy is also dependent on the depletion of CTLA-4 expressing regulatory T cells [14, 15]. However, Sharma et al. [16] found that anti-CTLA-4 treatment does not deplete Tregs in several human cancer types, suggesting there may be a discrepancy in anti-CTLA-4 response between mouse and human tumors. Therefore, attempts to understand the mechanism of action of anti-CTLA-4 antibodies could be improved by computational approaches that can identify biology shared by mice and humans and point to additional cell types beyond T cells that may mediate anti-CTLA-4 therapeutic efficacy.

**Fig. 1:**
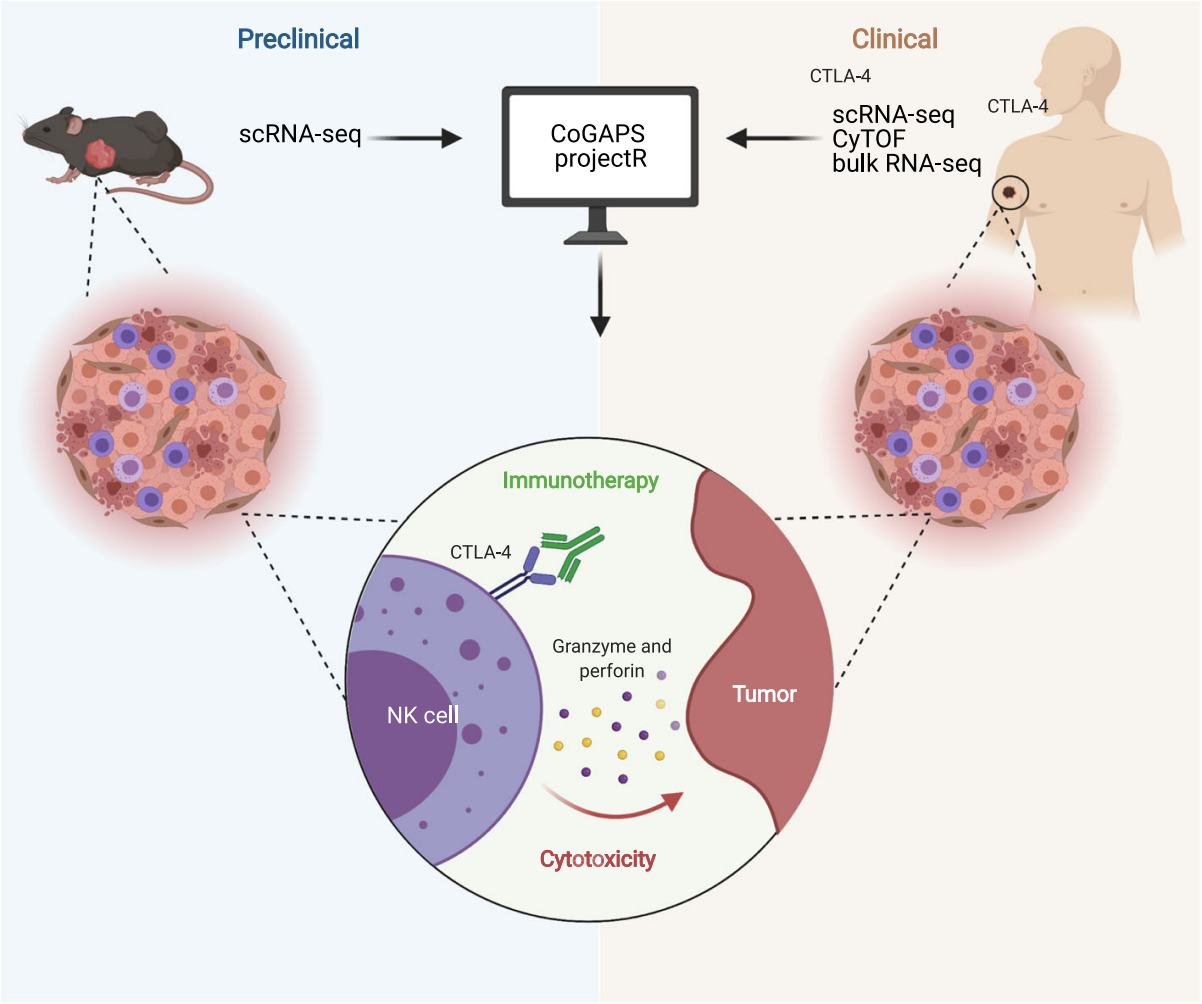
Graphical summary. Visual summary of the computational workflow, data types (scRNA-seq, CyTOF, or bulk RNA-seq), and sources (preclinical or clinical) used to identify conserved responses to immunotherapy. In response to anti-CTLA-4 therapy, we detect natural killer cell activation in mice and human tumors and demonstrate that human natural killer cells express CTLA-4 and bind anti-CTLA-4 at the cell surface

Altogether, this study provides an application of transfer learning to enable preclinical to clinical evaluation of cellular pathways associated with anti-CTLA-4 treatment. Using scRNA-seq data from Gubin et al. [4], we show that CoGAPS is able to detect robust transcriptional signatures associated with anti-CTLA-4 treatment (Fig. 1). The signature most associated with anti-CTLA-4-treated tumors reflected NK cell activation. We use projectR to confirm the association of this signature with positive clinical outcomes in datasets generated from distinct modalities that include bulk RNA-seq, mass cytometry, and scRNA-seq. This analysis identifies NK cell activation in anti-CTLA-4-treated human tumors that had not been described previously. We confirm our computational findings with complementary molecular techniques to begin to elucidate how NK cells activate in response to anti-CTLA-4 treatment. These analyses yield novel insights into the role of NK cells in anti-CTLA-4 efficacy and represent a general strategy for the study of shared tumor biology across datasets derived from different tumor types, treatment groups, sequencing platforms, and species.

## Methods

### Data collection

In this study, we used three public scRNA-seq datasets that were downloaded from NCBI’s Gene Expression Omnibus (GEO). For CoGAPS analysis on preclinical immunotherapy samples, we used the dataset from Gubin et al. (accession number GSE119352) [4]. This dataset contains ~ 15,000 flow-sorted CD45+ intratumoral cells from mouse sarcomas that were collected during treatment with either control monoclonal antibody, anti-CTLA-4, anti-PD-1, or combination anti-CTLA-4 and anti-PD-1 acquired with the 10x Genomics Chromium platform, using v1 chemistry. Associations between CoGAPS signatures and immunotherapy treatment were confirmed by transfer learning using paired mass cytometry data from Gubin et al. [4], which was downloaded from the FLOW Repository (FR-FCM-ZYPM) and processed using the R package cytofkit version 0.99.0.

For transfer learning to human samples, we used two scRNA-seq datasets of intratumoral immune cells from metastatic melanoma patients. To first test the relationship between our preclinical CoGAPS patterns and clinical outcome, we used the dataset from Sade-Feldman et al. (accession number GSE120575) [5]. This dataset contains ~ 16,000 flow-sorted CD45+ intratumoral cells obtained from 48 human melanoma tumor biopsies from 32 patients at baseline or after treatment with either anti-CTLA-4, anti-PD-1, or combination anti-CTLA-4 and anti-PD-1. This data was acquired with Smart-seq2 [17].

Next, to confirm the observed relationship between our preclinical NK activation signature and response to anti-CTLA-4, we used the scRNA-seq dataset from de Andrade et al. (accession number GSE139249) [6]. This dataset contains ~ 40,000 flow-sorted NK cells from matched blood and tumor samples obtained from 5 patients with melanoma metastases. Two patients had an initial response to treatment with anti-CTLA-4 or anti-PD-1 with oncolytic virus. Two patients failed to respond to combination anti-CTLA-4 and anti-PD-1 or anti-PD-1. One patient was not treated with immunotherapy. This data was acquired with the 10x Genomics Chromium platform, using v2 chemistry.

In addition, bulk RNA-seq was downloaded from The Cancer Genome Atlas [18]. Normalized gene expression for 33 tumor types were obtained using the R/Bioconductor package TCGAbiolinks version 2.14.1 [19]. CIBERSORT scores for this data were obtained from Thorsson et al. [20].

These datasets were used for pattern discovery and transfer learning as described below.

### Dimensionality reduction and cell type identification

Cell type inference analyses were performed for the Gubin et al. [4] dataset with the standard Monocle3 workflow using package version 0.2.0 [21–23]. Dimensionality reduction and visualization for scRNA-seq data were performed using Uniform Manifold Approximation and Projection (UMAP) [24]. Briefly, the first 15 principal components were used as input into the reduce_dimension function. Canonical cell type marker genes as described in Gubin et al. [4] were used to annotate cells.

### Mouse pattern discovery and gene set analysis using CoGAPS

CoGAPS analysis was performed using the R/Bioconductor package CoGAPS version 3.5.8 [25] to analyze the mouse sarcoma dataset from Gubin et al. [4]. Genes with a standard deviation of zero were removed prior to analysis. The input for CoGAPS is a data matrix of single-cell data with genomic features by cells, a number of sets, and number of patterns to learn (nPatterns) on each of the sets of cells. Because single-cell data is large, CoGAPS is performed for random subsets of cells in the complete scRNA-seq data as determined by the number of sets used as an input parameter to the software. CoGAPS factorizes the input matrix into two related matrices containing the gene weights (the amplitude (A) matrix) and sample weights (the pattern (P) matrix) for each data subset, and then identifies a set of consensus patterns across the data subsets and re-learns the amplitude (A) matrix on the entire dataset. Because consensus patterns are learned across multiple sets, the final number of patterns may not match the input parameter of nPatterns. The log2 transformed count matrix of remaining genes across all samples was used as input to the CoGAPS function. Default parameters were used, except nIterations = 50,000, sparseOptimization = True, and nSets = 12. The input parameters for nPatterns were determined empirically, by testing over a range of dimensions. When the nPatterns input was set to 3, we obtained results that identified immune cell lineage. We reasoned that additional patterns could further identify biological processes in the data related to treatment. We initially tested 50 patterns; however, many of the patterns highlighted few cells, indicating an over-dimensionalization of the data. When nPatterns was set to 25, CoGAPS identified 21 consensus patterns, which separated immune cell types and cell states.

Genes highly associated with each pattern were identified by calculating the PatternMarker statistic [26], which takes the gene weights assigned by CoGAPS and returns those most associated with a particular pattern or set of patterns. The CalcCoGAPSStat function was used to identify pathways significantly enriched in each pattern for the MSigDB hallmark gene sets [27] and PanCancer Immune Profiling panel from NanoString Technologies. This function links each CoGAPS pattern to the activity of input gene sets using a *z*-score based statistic [28]. *p*-values obtained from pathway analysis were FDR adjusted with the Benjamini-Hochberg correction and FDR adjusted *p*-values below 0.05 were called statistically significant.

### Pseudotime analysis

To perform pseudotemporal ordering, the dataset was subset to relevant cell types and treatments based on the desired analysis. Due to the association between pattern 7 and activation state markers, we chose the most active terminus of the trajectory as the end state. Thus, the root node of the trajectory was assigned by identifying the region in the UMAP-dimensional reduction with low CoGAPS pattern 7 weights. Pseudotime values were assigned to cells using the order_cells function from the R package Monocle3 version 0.2.0 [21–23]. Genes with significant expression changes as a function of pseudotime were identified using the graph_test function, using a multiple-testing corrected *q*-value cutoff of 0.01.

### Construction of multivariate Cox proportional hazards models

TCGA normalized gene expression for 33 tumor types was used as input for transfer learning to relate CoGAPS immune signatures to clinical outcomes. Metadata from Liu et al. [29] was used for measures of overall survival and age at diagnosis for TCGA samples. Samples were restricted to those that were labeled as “Primary solid tumor” (*n* = 9113), and “Metastatic” (*n* = 394) in the “definition” column of the TCGA metadata, which resulted in 9507 total samples. Association between CoGAPS pattern weights and overall survival was analyzed using multivariate Cox proportional hazards regression models using the survival coxph function from the R package survival version 3.2-11 and *p* < 0.05 was used as threshold for significance.

### Correlation analysis

To compare the expression of CTLA-4 and CIBERSORT scores for various immune cell types across immunogenic solid tumors from TCGA, we calculated the Spearman correlation coefficients using the cor.test function in R.

### Transfer learning

To examine whether the mouse patterns corresponded to similar immunotherapy responses in human data, we used The R/Bioconductor package projectR version 1.0.0 [30] to project the expression matrix from several datasets into the CoGAPS pattern matrix [7]. The CoGAPS result object and the expression matrix from a human dataset is used as input to the projectR function. Homologous genes present in the mouse and human data were retained for projection. Genes without homologs in the human data were removed. ProjectR returns a new pattern matrix, which estimates the role of each pattern in each cell of the human dataset. This comparison of pattern across species usage enabled us to determine how each pattern defines features present in the human dataset (i.e., cell types and immune cell activation). 

### Pattern performance of predicting anti-CTLA-4 response

The projected pattern weight is a continuous range of values, instead of a binary outcome. Using the individual projected pattern weight for each cell and a binary response outcome to anti-CTLA-4, we performed ROC curve analysis using the ROCR package, version 1.0-7 to determine the true-positive rates versus false-positive rates of pattern 7 weights to classify response. The area under the ROC curve was used as the quality metric to determine the prediction performance.

### Cell lines and materials

All human NK cell lines (NK-92, NK-92-CD16v, NKL, YT and KHYG-1) were kindly provided by Dr. Kerry S. Campbell (Fox Chase Cancer Center, Philadelphia, PA). The NK-92-CD16v expressed GFP due to transduction with pBMN-IRES-EGFP containing the Fc^γ^RIIIA construct. All NK cell lines were cultured as previously described [31]. Fresh healthy donor NK cells were purchased from AllCells (PB012-P). These NK cells were positively selected from donor peripheral blood using CD56 positivity. Donor NK cell purity was 98–99%. Donor 3 and donor 4 were expanded using engineered antigen presenting cells (K562-4-1BB-mbIL-21) according to the protocol [32]. CTLA-4 overexpressing Jurkat cell line was generated using lentiviral transduction purchased from G&P BIosciences (Product ID LYV-CTLA4, SKU# LTV0710) which contained full length human CTLA-4 gene subcloned into lentiviral expression vector pLTC with an upstream CMV promoter with puromycin selection marker. Jurkat cells were transduced using millipore sigma’s spinoculation protocol. In brief, lentiviral particle solution was added to 2 × 10^6^ Jurkat cells at a final multiplicity of infection of 1, 5, and 10. Cells were centrifuged at 800×*g* for 30 min at 32 °C then resuspended in complete growth medium for 3 days. After 3 days, cells were resuspended in complete medium containing 5 μg/mL puromycin overnight for selection. Selection was performed twice.

### qRT-PCR

RNA was isolated using the PureLink RNA Mini Kit (Ambion). The RNA concentration was measured using NanoDrop 8000 (Thermo Fisher Scientific). cDNA was generated from 20 to 100 ng of RNA using the GoTaq 2-step RT-qPCR System (Promega). qPCR was performed with SYBR Green on a StepOnePlus real-time PCR system (Applied Biosystems). Gene expression was normalized to HPRT and analyzed using 1/DCt method with triplicates.

Primers used were the following:

CTLA-4: (F: CATGATGGGGAATGAGTTGACC; R: TCAGTCCTTGGATAGTGAGGTTC)

CD28: (F: CTATTTCCCGGACCTTCTAAGCC; R: GCGGGGAGTCATGTTCATGTA)

CD28H: (F: CCCTGCAAGAAGCCTCAAG; R: CCTTTGTCCACTTAACACGGAG)

HPRT: (F: GATTAGCGATGATGAACCAGGTT; R: CCTCCCATCTCCTTCATGACA)

### Western blot

Cells were lysed in boiling buffer with EDTA (Boston BioProducts) supplemented with 1X protease and 1% phosphatase inhibitor prepared following the manufacturer’s protocols (Sigma-Aldrich, Cat. No. 11697498001 and P5726). Cleared lysate concentrations were obtained by a DC Protein Assay (BioRad). Lysates 30–50 μg were run on SDS-PAGE gels and transferred to nitrocellulose membranes (GE Healthcare). Western blots were conducted using anti-CTLA-4/CD152 (LS-C193047, LSbio) at concentrations of 1:1000 diluted in 5% milk in PBST. Secondary antibody was anti-rabbit IgG, HRP linked (Cell Signaling) used at 1:1000. Chemiluminescent substrate (Pierce) was used for visualization.

### Flow cytometry

All cells were aliquoted into Eppendorf tubes, spun at 5000 rpm for 1 min at 4 °C, washed twice with HBSS (Fisher Scientific Cat. No. SH3058801), and resuspended in 50 μL of FACS buffer (PBS plus 1% BSA) and blocked with 1 μL human Fc block (BD Biosciences, 564219) for 20 min at 4 °C. Labeled antibodies were then added at the manufacturer’s recommended concentrations and incubated at 4 °C for 30 min, with vortexing at 15 min. Cells were then washed with FACS buffer twice and resuspended in FACS buffer or fixative (1% PFA in PBS). Flow antibodies included anti-human CD152 (CTLA-4) (BD Bioscience 555853), CD28 (Biolegend 302907), and CD28H (R&D Systems, cat#MAB83162). The CD152 antibody has previously been shown to adequately detect CTLA-4 expression on both human T and B cells (29). Samples were run in the Georgetown Lombardi Comprehensive Cancer Center Flow Cytometry & Cell Sorting Shared Resource using BD LSRFortessa. Analyses were performed using FlowJo (v10.4.1).

### Immunofluorescence

Ipilimumab was acquired from the Medstar Georgetown University Hospital. Ipilimumab was labeled with Dylight550 fluorophore using the Dylight550 Conjugation Kit (Fast)-Lightning-Link (abcam, ab201800). In short, ipilimumab was diluted from 5 to 2 mg/mL using sterile PBS. Human IgG (Jackson ImmunoResearch, 009-000-003) was diluted from 11 to 2 mg/mL using sterile PBS. One microliter of modifying reagent was added to 10 μL diluted ipilimumab and 10 μL diluted human IgG. Ten microliters antibody was then added to the conjugation mix and incubated at room temperature in the dark for approximately 6 h. One microliter of quencher reagent was added to the labeled ipilimumab and the antibody was stored in the dark at 4 °C. NK-92 and PANC-1 cells were collected and washed with cold PBS and brought to a final concentration of 1 × 10^6^ cells/mL in staining buffer (1% BSA in PBS) in 50 μL. Fifty microliters of labeled ipilimumab or human IgG was added to cells to yield a final concentration of 1 μg/mL antibody. Cells were incubated in the dark at 4 °C for 1 h. After incubation, cells were pelleted and washed three times with cold PBS. Cells were brought to a final concentration of 0.5 × 10^6^ cells/mL and 100 μL was immobilized on slides using cytospin (Cytospin 2, Shandon) for 5 min at 1000 rpm. Following immobilization cells were fixed with 4% PFA for 10 min at room temperature then washed three times with cold PBS. Coverslips were mounted using VectraSheild mounting media with DAPI and sealed using clear nailpolish and allowed to dry overnight in the dark. Analyses were performed with the Leica SP8 AOBS laser scanning confocal microscope.

### Cell surface biotinylation

Cell surface biotinylation of NK92, NKL, YT, and KHYG-1 cells was performed with the Pierce Cell Surface Protein Isolation kit (Thermo Scientific, cat#89881) according to the manufacturer’s protocol. In brief, 4 × 10^8^ cells were pelleted and washed with cold PBS then incubated with EZ-LINK Sulfo-NHS-SS-biotin for 30 min at 4 °C followed by the addition of a quenching solution. Another 1 × 10^6^ cells were collected and saved for total cell western blotting. Cells were lysed with lysis buffer (500 μL) containing the cOmplete protease inhibitor cocktail (Roche, cat# 11697498001). The biotinylated surface proteins were excluded with NeutrAvidin agarose gel (Pierce, 39001). Samples were diluted 50 μg in ultrapure water supplemented with 50 mM DTT. Lysates were subjected to Western blotting with the anti-CTLA-4 antibody described above.

### NK cell stimulation

Cell lines or expanded primary NK cells were stimulated with 100 U/mL IL-2 (NCI preclinical repository), 5 ng/mL IL-12 (R&D Systems, cat#219-IL-005), 10 ng/mL IL-15 (NCI preclinical repository), 50 ng/mL IL-18 (Invitrogen, cat#rcyec-hil18), or 500 U/mL IFNg (Sigma-Aldrich, cat# I3265) for 24 h. Cell pellets were collected and processed for rt-qPCR as described above. Cell lines or expanded primary NK cells were stimulated with 3 μg/mL CD28 activating antibody (Biolegend, cat#302933) for 24 h.

## Results

### CoGAPS identifies known molecular alterations in response to immunotherapy from scRNA-seq data

**Fig. 2:**
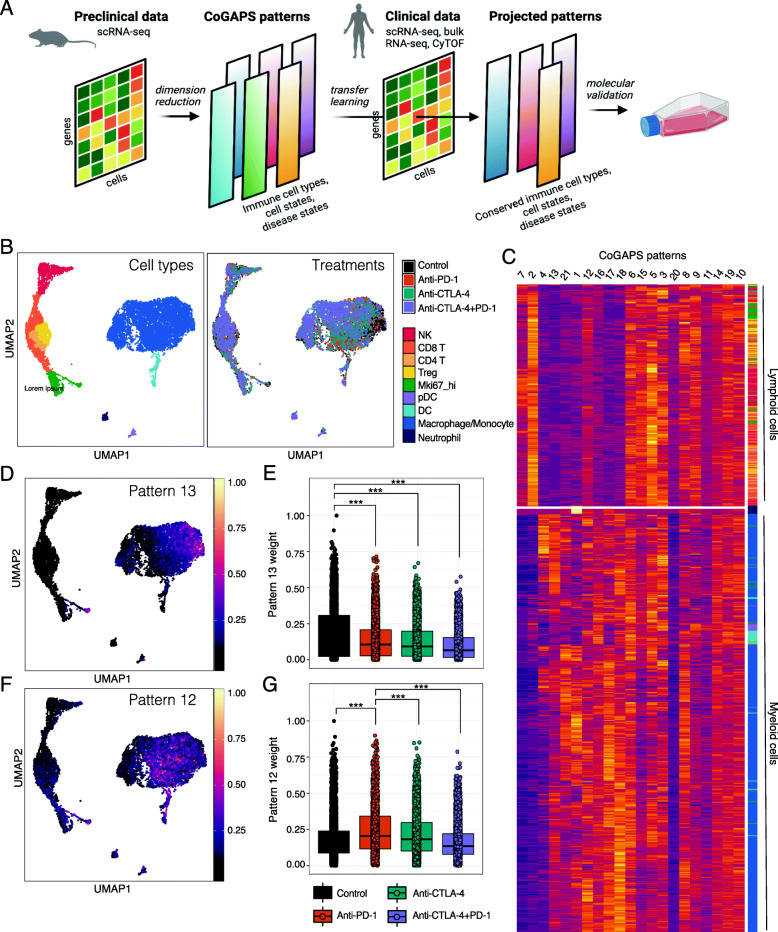
CoGAPS identifies gene signatures related to immune cell lineage and treatment response in mouse intratumoral immune cell scRNA-seq data. **A** Overview of the pipeline to relate preclinical and clinical mechanisms of action of therapy using transfer learning. First, CoGAPS, a non-negative matrix factorization algorithm is applied to scRNA-seq data of ICI-treated mouse tumors. Matrix factorization algorithms are unsupervised learning methods that can distinguish low-dimensional gene and cell features (latent spaces) associated with therapeutic responses without prior knowledge of gene regulation or cell type classification. Next, the transfer learning method projectR, is used to project the transcriptional signatures representing the latent spaces (or patterns) identified by CoGAPS into an independent dataset of human tumors treated. Finally, the cell weights representing relative usage of each pattern in the new human dataset can be computationally assessed for relationships to clinical outcomes and as the basis to prioritize candidates for experimental validation. **B** UMAP-dimension reduction of droplet-based scRNA-seq of intratumoral immune cells from ICI-treated mouse sarcomas [4]. Samples are colored by annotated cell types (left) and by treatment (right). **C** Hierarchical clustered heatmap of 21 CoGAPS patterns demonstrating segregation by immune cell lineage. Rows are individual cells, with row annotations designating cell type. Columns represent different CoGAPS patterns. **D** UMAP-dimension reduction colored by CoGAPS pattern 13 weights illustrates a cell type specific signature within the macrophages/monocytes. **E** Boxplot of pattern 13 weights in individual macrophage/monocyte cells, faceted by treatment group. Pattern 13 is associated with cells treated with control monoclonal antibody. Significant differences in mean pattern 7 weight between treatment groups are indicated by asterisks where *p*-values < 0.05 = *, < 0.01 = **, and < 0.001 = ***. **F** UMAP-dimension reduction colored by CoGAPS pattern 12 weights illustrates a cell type specific signature within the macrophages/monocytes. **G** Boxplot of pattern 12 weights in individual macrophage/monocyte cells, faceted by treatment group. Pattern 12 is associated with cells treated with anti-PD-1. Significant differences in mean pattern 7 weight between treatment groups are indicated by asterisks where *p*-values < 0.05 = *, < 0.01 = **, and < 0.001 = ***

Whereas human tumors have limited access for high-dimensional profiling, mouse models can be readily used to generate scRNA-seq data to study the tumor immune microenvironment under a variety of treatment conditions. Analysis of these data is then critical to determine biological processes associated with treatment perturbations, with unsupervised learning providing an opportunity for de novo discovery of cell state transitions related to therapy. To detect latent spaces (also called “patterns”) that represent transcriptional signatures across intratumoral immune cells during immunotherapy response, we used our non-negative matrix factorization (NMF) technique, CoGAPS (Fig. 2A) [25]. CoGAPS is an established approach to dissect transcriptional signatures that dictate cell type identity (i.e., NK vs. Treg) and cell state (i.e., activated vs. resting), aiding the evaluation of complex molecular alterations within the tumor immune microenvironment [33, 34]. By combining CoGAPS with projectR, a transfer learning approach, we can then quickly query for shared features across independent datasets across species (Fig. 2A) [7, 25].

To demonstrate the applicability of our pattern detection and transfer learning approach for cross-species analysis in the context of immunotherapy response, we first applied CoGAPS to identify transcriptional responses induced by ICIs in mouse tumors from a publicly available scRNA-seq dataset including more than 15,000 immune cells isolated from mouse sarcomas [4]. These tumors were treated with a control monoclonal antibody, anti-PD-1, anti-CTLA-4, or combination anti-PD-1 and anti-CTLA-4 antibodies (Fig. 2B). A critical challenge in applying matrix factorization algorithms such as CoGAPS to scRNA-seq analysis is selecting an appropriate dimensionality (i.e., number of patterns) to resolve biological features from the data [35]. Consistent with previous studies, running CoGAPS across multiple-dimensionalities revealed that different levels of biological complexity were captured at different dimensionalities [36]. For example, at low dimensionality (3 patterns), CoGAPS separated immune cells into myeloid and lymphoid lineages (Additional file 1: Fig. S1A). When dimensionality was increased to 21 patterns, the myeloid versus lymphoid lineage distinction was preserved and additional transcriptional signatures reflecting immune cell type and state were captured (Fig. 2C, Additional file 2: Table S1).

To identify specific attributes captured by each pattern, we performed gene set analysis using the gene weights for each pattern as input. We used the hallmark gene sets from the Molecular Signatures Database (MSigB) [27] and the PanCancer Immune Profiling gene panel from Nanostring Technologies to assess the enrichment of gene sets controlling well-defined biological processes. Gene set statistics for all patterns are provided in Additional file 2: Table S2. We found that several transcriptional signatures identified by CoGAPS were consistent with ICI-mediated changes previously described in the literature. For example, pattern 13 was enriched in macrophages/monocytes from progressing tumors treated with control monoclonal antibody (Fig. 2D, E). In contrast, pattern 12 was prevalent in macrophages/monocytes from tumors treated with anti-PD-1 (Fig. 2F, G). Macrophages are commonly divided into two subsets, pro-inflammatory anti-tumor M1 subtype and anti-inflammatory pro-tumor M2 subtype [37]. Consistent with this, pattern 13, which was enriched in control-treated tumors, reflected M2 macrophage polarization, which promotes tumor growth and metastasis (FDR adjusted *p*-value = 0.018, Additional file 2: Table S2). In contrast, pattern 12, which was enriched in anti-PD-1 treated tumors, reflected M1 macrophage polarization and interferon responses (FDR adjusted *p*-value = 0.046, Additional file 2: Table S2). This finding agrees with a recent study, which showed that anti-PD-1 treatment leads to a functional transition within the macrophage compartment towards an immunostimulatory M1 phenotype [38].

### CoGAPS analysis identifies a subset of activated NK cells in mouse tumors treated with anti-CTLA-4

**Fig. 3:**
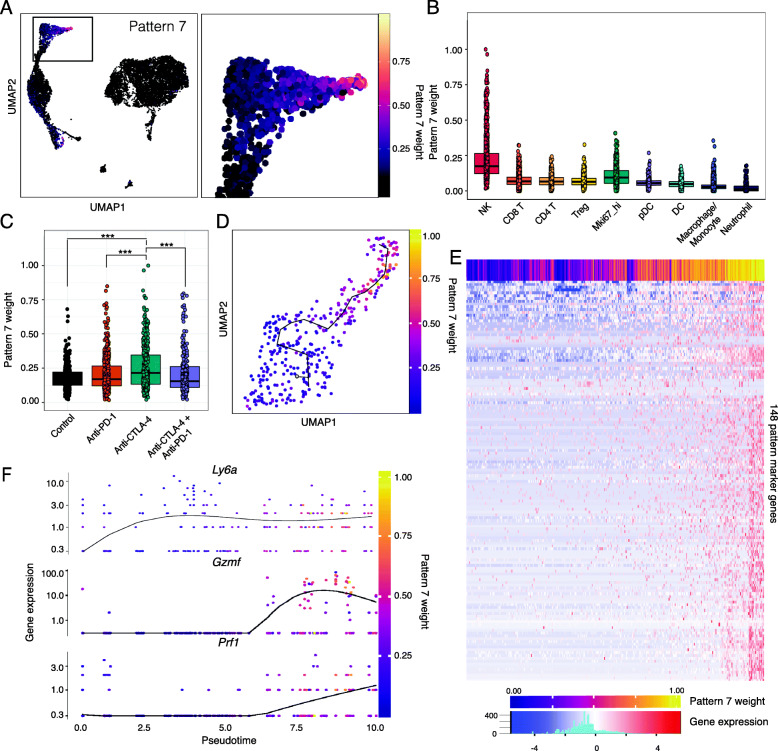
CoGAPS and pseudotime analysis reveals a dynamic state change in NK cells during ICI exposure in mouse scRNA-seq data. **A** UMAP-dimension reduction colored by CoGAPS pattern 7 weights across all cells (left) and magnified view (right) showing that pattern 7 marks a population of NK cells delineated in Fig. 2A. **B** Boxplot of pattern 7 weights across each immune cell type. Cells with high pattern 7 weights are observed only in NK cells. **C** Boxplot of pattern 7 weights in individual NK cells faceted by treatment group. Anti-CTLA-4-treated NK cells have increased pattern 7 weights compared to NK cells treated with other immunotherapies. Significant differences in mean pattern 7 weight between treatment groups are indicated by asterisks where *p*-values < 0.05 = *, < 0.01 = **, and < 0.001 = ***. **D** Pseudotemporal trajectory of anti-CTLA-4-treated NK cells colored by CoGAPS pattern 7 weight suggesting that anti-CTLA-4 treatment results in NK cell activation. **E** Heatmap of gene expression for 148 pattern markers with variable expression as a function of pseudotime. Columns are individual cells, and column annotation designates pattern 7 weight in each cell. Rows are differentially expressed pattern markers. **F** Gene expression of selected NK cell activation genes that are upregulated across pseudotime. Each dot represents a different cell and is colored by CoGAPS pattern 7 weight

In addition to the known transcriptional changes resulting from ICI treatment shown in Fig. 2, CoGAPS also identified a transcriptional signature that reflected a subset of activated NK cells—pattern 7 (Fig.3A, B). While tumors from each treatment group contained NK cells with elevated levels of pattern 7, there was a significant enrichment in NK cells from tumors treated with anti-CTLA-4 (Fig. 3C). To isolate the genes associated with this pattern, we used the CoGAPS PatternMarker statistic [26]. Instead of being based upon the CoGAPS gene weights, this statistic computes the unique association of genes with a particular pattern to isolate the specific set of genes associated with an inferred biological process to prioritize genes for validation. PatternMarker analysis identified 3195 genes associated with pattern 7. Gene set enrichment analysis on the CoGAPS result object revealed an upregulation of interferon-gamma and IL2-STAT5 gene sets in pattern 7, which are key pathways that govern cytotoxicity and maturation in NK cells (FDR adjusted *p*-value = 0.013, Additional file 2: Table S2) [39]. In addition, gene weights for pattern 7 were highest for markers of NK cell type and function (*NKG7*, *KLRK1*, *NCR1*, and *GZMB*) and negative for markers of T cells (*CD3D*, *CD3G*, *CD3E*, *CD4*, *CD8A*, and *CD8B1*) (Additional file 1: Fig. S1B).

The CoGAPS analysis suggested that pattern 7 identified NK cells undergoing a cell state change in response to therapy. To further confirm the CoGAPS inference of cell state transitions, we also performed pseudotime analysis on only the NK cells from tumors treated with anti-CTLA-4 [21]. While this analysis is not a time course of treatment response, trajectories learned from pseudotime analysis have been shown to enable a quantitative estimation of cellular progression through cell state transitions associated with dynamic biological processes. The pseudotemporal ordering of anti-CTLA-4-treated NK cells showed a sequential progression in cellular trajectory (Fig.3D). This pseudotime trajectory was highly correlated with the pattern 7 weight identified in each cell (0.71 spearman correlation). Notably, the trajectory revealed a single transition state in NK cells as a result of anti-CTLA-4 treatment, with individual cells having transcriptional profiles that reflect various points along the trajectory.

Regression analysis to detect genes significantly associated with changes in pseudotime identified 1968 genes at a *q*-value threshold of 0.01 in anti-CTLA-4-treated tumors (Table S3). We then looked for genes that were both significantly associated with pseudotime and patternMarkers of the CoGAPS pattern 7 to obtain a subset of 148 genes related to NK cell transitions with anti-CTLA-4 treatment (Fig. 3E). This analysis identified 148 genes, including markers of NK cell activation such as perforin, granzymes, and Ly6a [40] (Fig. 3F). These data support recent findings that NK cells within mouse tumors can be functionally modulated by ICI treatment [41, 42].

In their original study, Gubin et al. [4] used CyTOF, a mass spectrometry-based flow cytometry method to measure protein expression in parallel with their scRNA-seq. By CyTOF, they found that anti-CTLA-4-induced Granzyme B in a population of KLRG1+ NK cells independently from the scRNA-seq analysis. Still, the relationship between anti-CTLA-4 and NK cell activation in this subpopulation was not evaluated in that study. We hypothesized that immune cells from tumors treated with anti-CTLA-4 in the CyTOF data would have elevated levels of the transcriptional NK cell activation signature we detected in the scRNA-seq data. To test this hypothesis, we used our transfer learning method, projectR [30], to assess the CyTOF data for the 21 patterns identified by CoGAPS from scRNA-seq. As expected, we found that pattern 7 was highest in immune cells from anti-CTLA-4-treated tumors profiled by CyTOF (Additional file 1: Fig. S1C). These findings demonstrate that (1) CoGAPS identified transcriptional changes in response to immunotherapy, which is preserved at the protein and mRNA level and across technological platforms, (2) CoGAPS identified an NK cell activation signature in the scRNA-seq data that was missed by the traditional scRNA-seq analysis methods used in the original study, and (3) ProjectR is capable of identifying gene expression signatures present in both scRNA-seq and CyTOF data.

### Preclinical NK cell activation signature is associated with ipilimumab response in metastatic melanoma

To investigate the relevance of the NK cell activation signature (pattern 7) learned in the preclinical mouse model to immunotherapy responses in humans, we used our transfer learning method (projectR), to project two independent scRNA-seq datasets of ICI-treated metastatic melanoma patients [5, 6] into the 21 mouse patterns identified by CoGAPS. We selected melanoma datasets since ICI treatment is widely used in melanoma patients and because previous studies have shown that transcriptional signatures of NK cell infiltration correlate with improved clinical outcomes in melanoma [43]. First, we analyzed a scRNA-seq dataset of ~ 16,000 immune cells isolated from melanoma metastases. Patients in this study were treated with anti-PD-1, anti-CTLA-4, or combination anti-PD-1 and anti-CTLA-4 antibodies, and the biopsies used for scRNA-seq profiling were taken either before or during treatment [5]. Using the projected weights of each signature and treatment outcomes, we evaluated the association of each pattern with therapeutic response in humans. In pre-treatment biopsies, the NK cell activation signature was significantly higher in anti-CTLA-4 responsive tumors than non-responsive tumors (*p* < 1 × 10^−15^, Additional file 1: Fig. S2A). This is consistent with our initial finding that NK cell activation was enriched in mouse tumors treated with anti-CTLA-4.

**Fig. 4:**
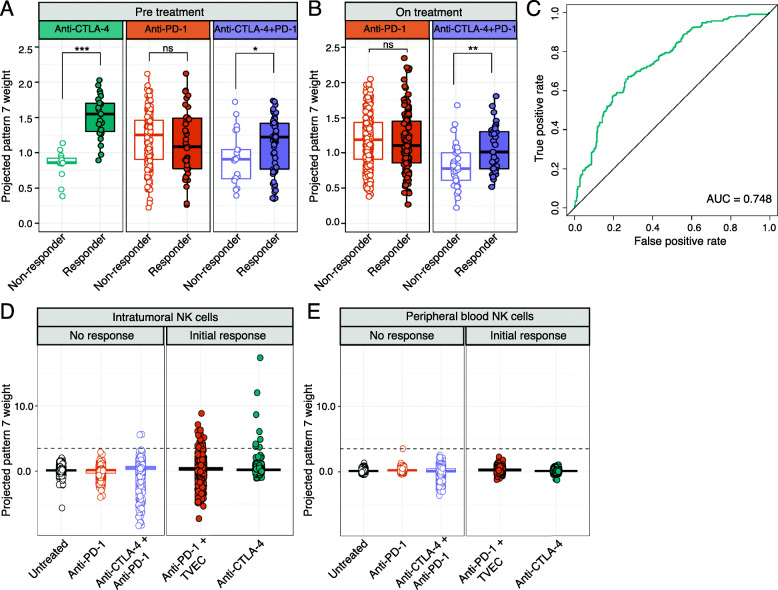
ProjectR recovers conserved immunotherapy response in intratumoral NK cells from independent human melanoma scRNA-seq datasets. **A** Box plot of projected pattern 7 weights across intratumoral NK cells from metastatic melanoma patients prior to ICI treatment [5]. Cells are colored by therapy and separated by patient response. Increased pattern 7 is significantly associated with NK cells from patients responsive to anti-CTLA-4 or combined anti-CTLA-4 and anti-PD-1. Significant differences in mean pattern 7 weight between treatment groups are indicated by asterisks where *p*-values < 0.05 = *, < 0.01 = **, and < 0.001 = ***. **B** Box plot of projected pattern 7 weights across intratumoral NK cells from metastatic melanoma patients after treatment with ICI. Cells are colored by therapy and separated by patient response. Increased pattern 7 is associated with NK cells from patients responsive to combination anti-CTLA-4 + anti-PD-1. Significant differences in mean pattern 7 weight between treatment groups are indicated by asterisks where *p*-values < 0.05 = *, < 0.01 = **, and < 0.001 = ***. **C** ROC curve for the performance of pattern 7 weights in predicting response to anti-CTLA-4 prior to the administration of treatment. **D** Box plot of projected pattern 7 weights across flow-sorted intratumoral NK cells from metastatic melanoma tumors that were unresponsive ICI (intrinsic resistance) or developed acquired resistance after a period of initial response [6]. The dashed line indicates the average maximum value for pattern 7 across treatment groups. NK cells with elevated pattern 7 weights are seen in patients that had an initial response to ICI, with the highest observed weights from a patient that responded to anti-CTLA-4. **E** Box plot of projected pattern 7 weights across NK cells isolated from peripheral blood of metastatic melanoma patients that had no response to ICI (intrinsic resistance) or developed acquired resistance after a period of initial response. The dashed line indicates the average maximum value for pattern 7 from intratumoral NK cells across treatment groups. Elevated pattern 7 weights are not detected in circulating NK cells, regardless of response.

Previous scRNA-seq studies that have identified subpopulations of T cells that express transcripts linked to the cytotoxic function of NK cells, such as NKT cells [44, 45]. Consistent with these findings, we observed that cells expressing canonical NK marker genes (*NCR1* and *FCGR3A*) were intermixed with cells expressing T cell marker genes (CD3D) in the lymphocyte cluster (Additional file 1: Fig. S2B). In addition to showing that pattern 7 is specific for NK cell genes (Additional file 1: Fig. S1B), to further ensure that T and NKT cells were excluded from analysis and specifically focus on human NK cells, we performed a gene expression gating strategy that required the expression of several transcripts related to NK cell function (*NCR1*, *NKG7*, and *FCGR3A*) and a lack of the T cell transcripts (*CD4*, *CD3D*, and *CD3G)*. Gating for NK cells confirmed that the NK cell activation signature was enriched in intratumoral NK cells isolated from anti-CTLA-4 responsive tumors (Fig. 4A, *p* < 1 × 10^−8^). Because cells were obtained from tumor biopsies prior to the administration of anti-CTLA-4 treatment, this finding suggests that cytotoxic NK cell infiltration could be predictive of anti-CTLA-4 response. In patients treated with anti-PD-1, there was no significant difference in the NK cell activation signature between responders and non-responders regardless of whether biopsies were taken before (Fig. 4A, *p* > 0.05) or during (Fig. 4B, *p* > 0.05) treatment. In contrast, the NK cell activation signature was significantly enriched in tumors responsive to combination anti-CTLA-4 and anti-PD-1 taken before (Fig. 4A, *p* < 0.05) and during (Fig. 4B, *p* < 0.01) treatment. Using receiver operating characteristic curve (ROC) analysis, we found that the NK cell activation signature had a moderate ability to classify anti-CTLA-4 response (Fig. 4C, AUC = 0.748), suggesting that the NK activation signature has the potential utility to predict responsiveness to anti-CTLA-4 from pre-treatment tumor biopsies. These findings indicate that the presence of active NK cells within tumors is important to the clinical usage and success of anti-CTLA-4 therapies.

Although ICI therapy can lead to durable responses in patients with metastatic melanoma, intrinsic and acquired resistance remain major causes of mortality [46]. To examine the relationship between NK cell activation and mechanisms of therapeutic resistance, we next projected the transcriptional patterns into a scRNA-seq dataset of NK cells isolated by flow cytometry from matched melanoma metastatic lesions and blood samples of patients that had progressed after immunotherapy [6]. This dataset included two patients that had an initial response to ICI (acquired resistance), two patients that failed to respond to ICI (intrinsic resistance), and one patient that was not given ICI (untreated). We found high levels of the NK cell activation signature in a subset of intratumoral NK cells from the two patients who had an initial response to ICI (Fig. 4D). Consistent with our results which indicate that the NK cell activation signature is enriched in anti-CTLA-4 responsive tumors, the highest levels of the NK cell activation signature were found in NK cells from the patient responsive to anti-CTLA-4 (ipilimumab). Elevated NK cell activation signature was also found in the patient responsive to combination treatment with anti-PD-1 and oncolytic virus (pembrolizumab + TVEC). Notably, these observations were specific to intratumoral NK cells, as the NK cell activation signature was detected only at very low levels in NK cells isolated from matched peripheral blood samples (Fig. 4E). This result indicates that anti-CTLA-4 treatment leads to NK cell activation specifically within the tumor microenvironment in humans, consistent with observations in mice [42].

### Human NK cells express CTLA-4, which is bound by ipilimumab

**Fig. 5:**
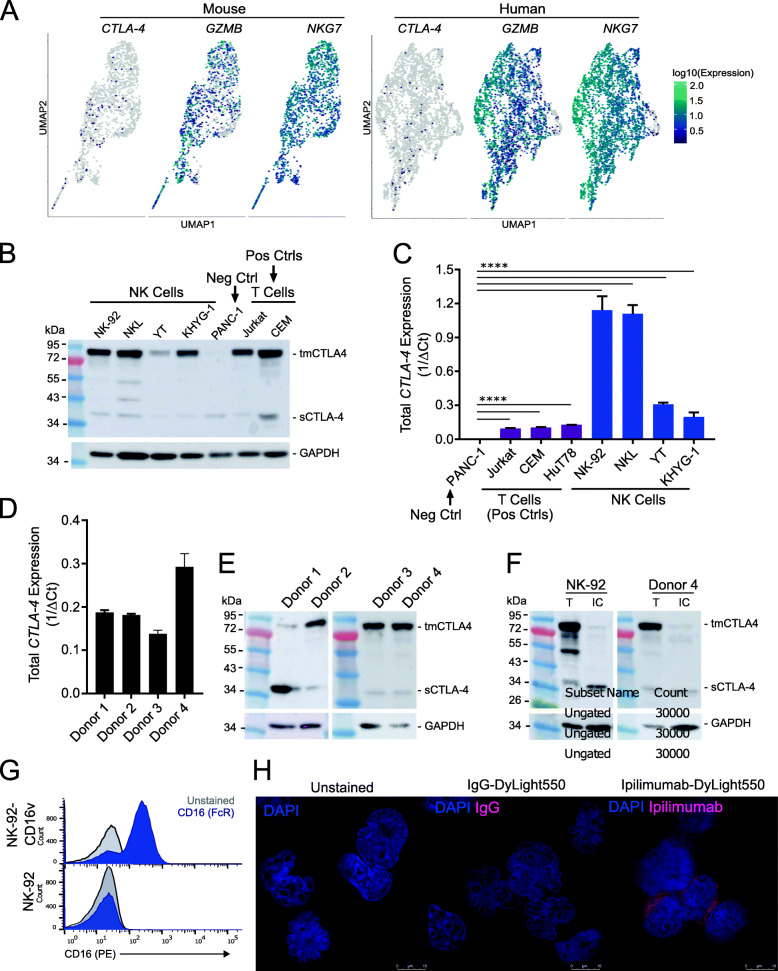
CTLA-4 is expressed by both human NK cell lines and healthy human donor-derived NK cells. **A** UMAP-dimension reduction with cells colored by single-cell gene expression for *CTLA-4* and representative immune activation genes in mouse (left) and human (right) intratumoral NK cells. The pattern of *CTLA-4* expression is consistent with the reduced ability of scRNA-seq to capture low to moderately expressed genes. **B** Western blot demonstrating CTLA-4 expression in human NK cell lines. Representative of two independent experiments. **C** Quantitative real-time PCR (qRT-PCR) analysis of total *CTLA-4* expression (both isoforms) in a CTLA-4 null line (PANC-1), T cell lines (Jurkat, CEM, HuT78), and NK cell lines (NK92, NKL, YT, KHYG-1). *p*-value < 0.001 = **** as determined by unpaired, two-tailed t-test. **D** qRT-PCR demonstrating *CTLA-4* expression in CD56+ selected ex vivo unstimulated NK cells derived from healthy human donors. **E** Western blot of CTLA-4 expression in CD56+ selected ex vivo unstimulated NK cells derived from healthy human donors. **F** Western blot of total protein (T) and intracellular (IC) protein isolated from human NK cell line NK-92 and unstimulated primary human NK cells using cell surface protein biotinylation for exclusion of surface proteins demonstrating surface expression of CTLA-4 dimers and intracellular expression of CTLA-4 monomers. **G** Flow cytometry demonstrating NK-92 does not express antibody receptor CD16. Positive control was the NK-92 line that had been transfected with a CD16 expressing plasmid, NK-92-CD16v. **H** Immunofluorescent images of NK-92 cells stained with Dylight550-labeled ipilimumab demonstrating that ipilimumab binds to the NK cell surface. Blue staining indicates DAPI. Shown are representative images of a single field of view taken via confocal microscopy (magnification, 63×; zoom, 3×)

CTLA-4 is an important regulator of T cells, and there is growing evidence suggesting that CTLA-4 regulates other human immune cell types, including B cells [47, 48], monocytes [49], and dendritic cells [50]. While our computational analysis suggests a functional role of CTLA-4 in human NK cells, expression of CTLA-4 in human NK cells is controversial in the literature; most studies indicate that human NK cells do not express CTLA-4 [42, 51–53]. Our computational association of the intratumoral NK cell activation in response to anti-CTLA-4 treatment suggests that NK cell activity may be modulated directly by CTLA-4 treatment and that CTLA-4 may function as an NK cell immune checkpoint—similar to its role in T cells. To investigate this possibility, we used scRNA-seq data to assess the expression of *CTLA-4* transcripts in NK cells and the relationship between CTLA-4 expression and expression of NK cell activation markers. Indeed, we found clusters of intratumoral NK cells from mice and humans that express *CTLA-4* and markers of NK cell activation, including *GZMB* and *NKG7* (Fig. 5A). Given that *CTLA-4* transcripts were detectable in a handful of NK cells, *CTLA-4* may be expressed at low to moderate levels and result in poor capture efficiency during scRNA-seq [54]. These technical limitations make the use of in vitro techniques necessary to validate computational findings. Therefore, we turned to molecular biology to further investigate the transcriptional signature of NK cell activation.

To confirm that human NK cells express CTLA-4, we directly tested four human NK cell lines (NK-92, NKL, YT, and KHYG-1) for CTLA-4 expression at the mRNA and protein level and compared to a negative control CTLA4-null cell line (PANC-1) and positive control T cell lines (Jurkat, CEM, HuT78). While all four cell lines appeared negative for CTLA-4 by flow cytometry (Additional file 1: Fig. S2A), all NK cell lines revealed robust CTLA-4 expression determined by western blot and qRT-PCR (Fig. 5B, C). CTLA-4 is known to be expressed on several tumor-derived human cell lines [55, 56]. To exclude the possibility that this observation was specific to malignant NK cells, we assessed CTLA-4 expression in unstimulated ex vivo CD56+ NK cells isolated from healthy human donor PBMCs. Consistent with the results in NK cell lines, CTLA-4 was undetectable by flow cytometry (Additional file 1: Fig. S2B). However, western blot and rt-qPCR confirmed that NK cells from each donor constitutively expressed CTLA-4 (Fig. 5D, E).

Since the western blots of both the positive control T cell lines and NK cells shows two bands—one representing the ~ 95 kDa dimer that is surface expressed and one representing the ~ 30 kDa monomer that is intracellular—we hypothesized that antibody-specific limitations were precluding successful detection of CTLA-4 on the NK cell surface by flow cytometry. We, therefore, turned to an antibody-independent means of detecting surface expression—surface protein biotinylation—to confirm that NK cells express CTLA-4 on the surface. We biotinylated cell surface proteins and then excluded them from the cell lysate via magnetic separation. Using the NK cell line NK92 and healthy donor NK cells, we determined that CTLA-4 dimers and monomers are present in total cell lysate, but the CTLA-4 dimers are absent from the intracellular protein lysate, confirming that NK cells express CTLA-4 dimers on their surface (Fig. 5F).

In T cells, CTLA-4 competes with co-stimulatory receptor CD28 for B7 ligands. When CTLA-4 outcompetes CD28 for B7 binding, it prevents CD28 co-stimulatory signaling and instead provides inhibitory signaling. Anti-CTLA-4 treatment results in T cell activation by inhibiting the inhibitor, by blocking CTLA-4-B7 interactions and promoting CD28-B7 interactions. To determine if CTLA-4 could be functioning similarly in NK cells, we tested NK cells for CD28 and CD28H expression. Consistent with previous reports, we found that some NK cell lines and donor NK cells expressed CD28 and CD28H [57] by flow cytometry and qRT-PCR (Additional file 1: Fig. S4). Thus, human NK cells express both CTLA-4 and CD28, supporting a similar role for these receptors in T cells and NK cells.

### Ipilimumab binds to CTLA-4 expressed on the NK cell surface independent of CD16

We next wanted to determine if the anti-CTLA-4 antibody, ipilimumab, was capable of binding to CTLA-4 expressed on the NK cell surface. To do so, we fluorescently labeled anti-CTLA-4 (Ipilimumab) to probe for ipilimumab binding to the NK cell surface by immunofluorescence microscopy. One potential complication is a nonspecific binding of ipilimumab to NK cells. Human NK cells express antibody receptors (e.g., Fc receptor CD16) which can bind to the constant region of an antibody regardless of the antibody’s specificity [58].. To exclude the possibility of nonspecific ipilimumab-NK cell interactions, we used the human NK cell line NK-92, which lacks generic antibody receptors (i.e., CD16) (Fig. 5G). Immunofluorescence imaging demonstrated that fluorescently labeled anti-CTLA-4, but not the IgG control, was capable of binding to NK-92 through recognition of CTLA-4 on the cell surface (Fig. 5H). The specificity of the stain was confirmed using the CTLA-4 null line PANC-1 (Additional file 1: Fig. S2E). We saw abundant surface expression of CTLA-4 by immunofluorescence, confirming the results shown in Fig. 5F. To the best of our knowledge, this is the first demonstration that anti-CTLA-4 (ipilimumab) can directly interact with human NK cells via a CD16-independent mechanism.

### NK cell activation regulates CTLA-4 expression

**Fig. 6:**
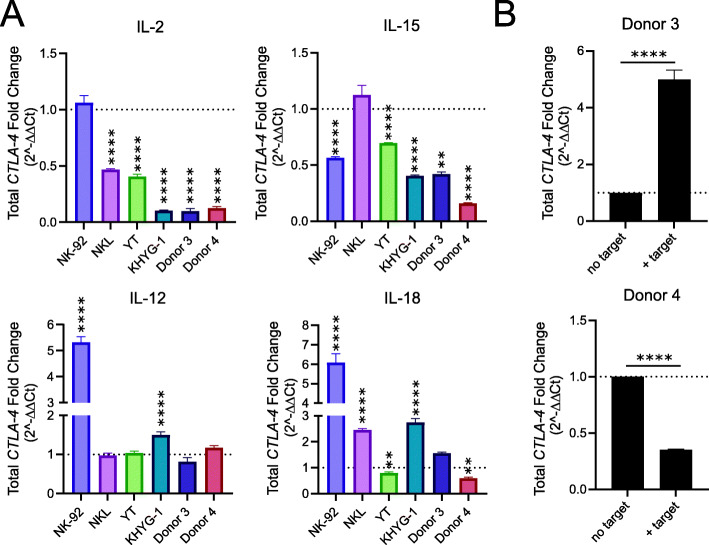
NK cell activation regulates CTLA-4 expression. **A** Effect of 24-h stimulation with IL-2, IL-12, IL-15, and IL-18 on NK cell CTLA-4 expression as determined by qRT-PCR (*n* = 3 for NK cell lines, 2 for donor NK cells; *p*-values < 0.01 = ** and < 0.0001 = **** when comparing ΔCt for that cell after exposure to cytokine to that cell line unexposed using an unpaired two-tailed *t*-test). **B** Effect of target cell exposure (K562-4-1BB-mbIL-21) on NK cell CTLA-4 expression as determined by qRT-PCR (*n* = 3, *p*-value < 0.0001 = **** when comparing ΔCt using an unpaired two-tailed t-test)

In T cells, CTLA-4 expression is modulated in response to T cell activation via CD28 and T cell receptor signaling [59]. To investigate if in vitro NK cell activation would similarly modify CTLA-4 expression in NK cells, we exposed NK cells to a variety of cytokines (IL-2, IL-12, IL-15, IL-18) that activate NK cells and alter NK cell expression of other immune checkpoints (i.e., PD-1) [60, 61] (Fig. 6A). Human NK cells, with the exception of NK cell line NK-92, had a drastic reduction in CTLA-4 after 24-h exposure to IL-2. IL-15 also caused a reduction in CTLA-4 expression in all NK cells tested except NKL. Alternatively, IL-12 and IL-18 increased CTLA-4 expression in a subset of NK cell lines, including primary donor NK cells. The variability in CTLA-4 expression in response to cytokine stimulation may be attributed to intrinsic differences in the NK cell lines, which can alter their response to certain stimuli. For instance, the NK92 cell line does not express any of the KIR family of inhibitory receptors; therefore, this cell line is thought to be hyper-sensitive to cell-mediated activation [62].

Target cell recognition is another means to activate NK cells. Since cytokine-activated and target cell-activated NK cells have distinct transcriptional phenotypes [63], we also investigated target cell-mediated NK cell activation on NK cell CTLA-4 expression by exposing NK cells to engineered target cells (K562-4-1BB-mbIL-21 cells) (Fig. 6B). Although we saw divergent responses in the primary NK cells from two donors, target cell exposure clearly modulated CTLA-4 expression. These data demonstrate that although responses are variable, human NK cell activation, via cytokine and target cell stimulation, alters NK cell expression of CTLA-4. Combined with the observation that anti-CTLA-4 antibodies bind human NK cells, these results suggest CTLA-4 may be an NK cell checkpoint and drive the computationally identified signature of NK cell activation in anti-CTLA-4 responsive tumors. Taken together, these results confirm the utility of CoGAPS and projectR to identify conserved biological processes between preclinical models and human patients that contribute to clinical outcomes.

### Preclinical NK cell activation signature is associated with overall survival in metastatic melanoma patients

**Fig. 7:**
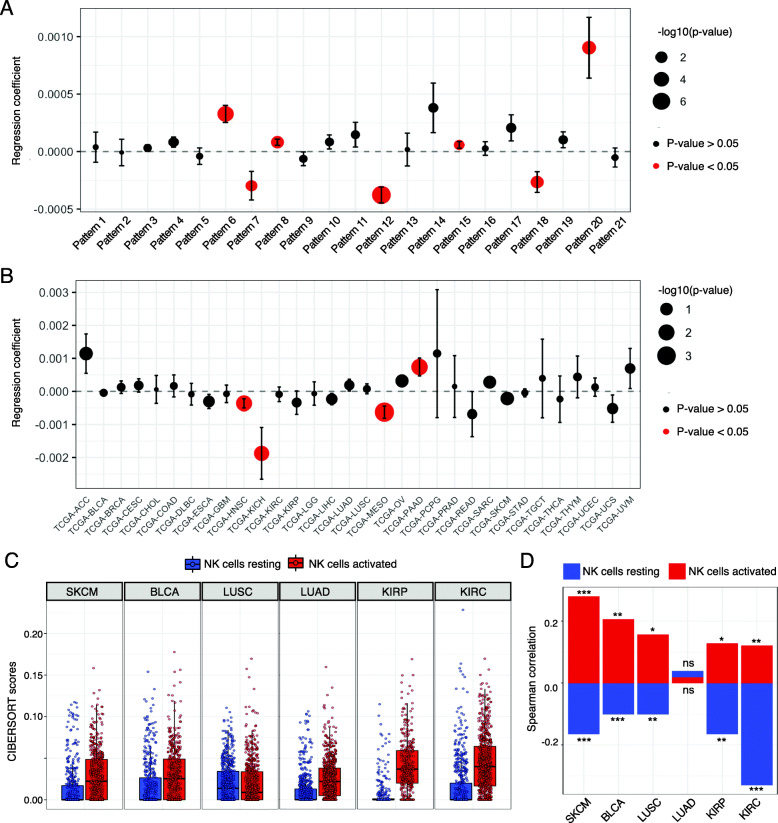
Preclinical NK activation signature is associated with overall survival in human melanoma. **A** Coefficients of an age-adjusted multivariate Cox proportional hazards regression model that relates CoGAPS patterns and overall survival in metastatic melanoma lesions from TCGA. Point size scaled to the coefficient’s *p*-value. Red points indicate patterns with significant coefficients. A positive coefficient indicates a worse overall survival and a negative coefficient indicates a better prognosis for the associated variable. **B** Coefficients of an age-adjusted multivariate Cox proportional hazards regression model that relates CoGAPS pattern 7 and overall survival across 32 primary tumor types from TCGA. Point size scaled to the coefficient’s *p*-value. Red points indicate patterns with significant coefficients. **C** Boxplot of CIBERSORT scores estimating the abundance of resting and activated NK cells from TCGA RNA-seq data by tumor subtype in TCGA. **D** Bar plot of Spearman correlation coefficients between CTLA-4 and CIBERSORT cell type score for immunogenic cancers. CTLA-4 expression is positively correlated with estimation of activated NK cells from TCGA RNA-seq data. Significant correlations for NK scores and CTLA-4 expression are indicated by asterisks where *p*-values < 0.05 = *, < 0.01 = **, and < 0.001 = ***

We hypothesized that the CoGAPS identified NK cell activation signature might be detectable in untreated tumors that naturally elicit an anti-tumor NK cell response, such as melanoma metastases [6]. In addition to the ability to relate biological processes across species, our transfer learning approach can be used to compare across sequencing platforms. Therefore, to investigate if NK cell activation was associated with clinical outcomes in untreated cancer patients, we used projectR to project bulk RNA-seq data from TCGA of 9507 human tumors representing 32 solid tumor types into the 21 CoGAPS patterns originally identified in scRNA-seq [18]. An association between CoGAPS pattern weight and overall survival was determined using a multivariate Cox proportional hazards model, adjusted for age. In melanoma, pattern 7 weight in metastatic lesions (*n* = 368) was associated with a longer overall survival (Fig. 7A, HR = 0.99, *p* = 0.017). Pattern 7 weight in primary melanoma lesions (*n* = 103) was not associated with any statistically significant difference in overall survival (Additional file 1: Fig. S5). These results show that NK cell activation is significantly associated with overall survival in untreated metastatic melanoma patients. The association between our NK cell activation pattern and clinical outcomes in metastatic lesions is consistent with the role of NK cells in controlling cancer progression and metastasis [64].

When fitting separate Cox proportional hazards models by cancer type across all primary tumor types and adjusting for age, head and neck squamous cell carcinoma (HNSCC), kidney chromophobe (KICH), and mesothelioma (MESO) showed a significant association between pattern 7 weight and overall survival (Fig. 7B). Consistent with this, several studies have similarly found an association between infiltrating NK cell abundance or function and overall survival in solid tumor types, including HNSCC [65–70]. Interestingly, pattern 7 weight in primary pancreatic adenocarcinoma (PAAD) was associated with a significantly worse overall survival. Notably, studies of the association between NK cells and disease prognosis in PAAD have had inconsistent findings [71–74]. The association between pattern 7 and worse overall survival in PDAC may be driven by abnormal NK activation or dysregulation of the innate immune system within some lesions. As there is no universal cell type marker to define NK cells and different subsets express standard marker genes differently, studies investigating the relationship between NK cell infiltration and overall survival are limited in their ability to assess the relationship between overall survival and the abundance of functional subpopulations [70]. Bulk RNA-seq similarly suffers from a limited ability to delineate cell types and states from aggregate transcriptional data. In contrast, our results demonstrate we can computationally project transcriptional signatures identified from scRNA-seq data into bulk RNA-seq data to rapidly detect immune cell states shared between distinct species and data modalities. In addition, these results confirm that NK cell activation is associated with overall survival in metastatic melanoma [43].

### CTLA-4 expression is positively correlated with the infiltration of active NK cells in immunogenic human tumors

Given that the NK cell activation signature was enriched in anti-CTLA-4-treated mouse tumors, we hypothesized that there may be a correlation between CTLA-4 expression and intratumoral NK cell content. To explore this hypothesis, we used bulk RNA-seq data from TCGA then applied CIBERSORT, a widely used computational approach that infers immune cell content from bulk RNA-seq data [75]. In this analysis, we assessed six immunogenic solid tumor types: skin cutaneous melanoma (SKCM), kidney renal clear cell carcinoma (KIRC), cervical kidney renal papillary cell carcinoma (KIRP), squamous cell carcinoma of the lung (LUSC), lung adenocarcinoma (LUAD), and bladder carcinoma (BLCA). When running CIBERSORT, we used the LM22 signature matrix designed by Newman et al. [75] to estimate the relative fraction of 22 immune cell types within input mixture samples, including an estimation of resting and activated NK cell proportions (Fig. 7C). Correlation analysis across the 21 CoGAPS patterns for the genes present in both the CoGAPS amplitude matrix and the LM22 signature matrix (*n* = 391) found that pattern 7 had the highest correlation (Pearson = 0.497) to the CIBERSORT NK cell activation signature (Table S4), further supporting the association between pattern 7 and NK cell activation. Correlation analysis between CTLA-4 expression and CIBERSORT cell type estimation revealed that the direction of correlation in NK cells was dependent upon the activation state (Fig. 7D, Table S5). Across several tumor types, the proportion of activated NK cells was positively correlated with CTLA-4 expression, while the proportion of resting NK cells was negatively correlated. CTLA-4 expression was negatively correlated with estimated proportions of resting NK cells in SKCM (*p* < 1 × 10^−4^), BLCA (*p* < 1 × 10^−3^), LUSC (*p* < 1 × 10^−2^), KIRP (*p* < 1 × 10^−2^), and KIRC (*p* < 1 × 10^−9^). On the other hand, estimated proportions of activated NK cells were positively correlated with CTLA-4 expression in SKCM (*p* < 1 × 10^−6^), BLCA (*p* < 1 × 10^−2^), LUSC (*p* < 0.05), KIRP (*p* < 0.05), and KIRC (*p* < 1 × 10^−2^). As expected, CTLA-4 expression was also positively correlated with the estimated proportions of regulatory T cells (Tregs) in each tumor type (Table S5). This analysis complements our experimental results and further supports a relationship between NK cell activation, CTLA-4 expression, and clinical outcomes in human tumors.

## Discussion

In this application of matrix factorization and transfer learning to cancer immunotherapy, we demonstrate both computationally and experimentally that this approach can elucidate complex immunotherapy responses from scRNA-seq data that are conserved across species. Specifically, we show that our matrix factorization approach (CoGAPS) detected a signature of intratumoral NK cell activation in anti-CTLA-4-treated mice which our transfer learning method (projectR) associated with positive clinical outcomes in metastatic melanoma. We interrogate and validate this NK cell activation signature in several datasets, including proteomics (CyTOF), bulk RNA-seq (TCGA), and additional scRNA-seq. Ultimately, the application of these computational techniques identified novel biology—that human NK cells express CTLA-4, bind anti-CTLA-4 (ipilimumab), and NK cell activation associates with anti-CTLA-4 activity in human tumors.

Both CoGAPS and projectR offer unique advantages to interpreting complex tumor immune cell scRNA-seq data. For instance, traditional clustering methods such as those employed by Gubin et al. [4] group cells according to transcriptional signatures that reflect cell type. However, a single cell’s transcriptional profile represents more than just cell type, encompassing additional cellular processes such as activation, exhaustion, and cell signaling, which are not necessarily captured by traditional clustering approaches. Identifying these cellular processes is particularly important when studying immune cells within the tumor microenvironment, where cells may undergo stimulation or dysregulation. In the scRNA-seq data, Gubin et al. [4] did not detect NK cell activation in anti-CTLA-4-treated tumors; however, their subsequent CyTOF analysis revealed prominent upregulation of NK cell granzyme expression specific to anti-CTLA-4 treatment [4]. In contrast, our matrix factorization method, CoGAPS, was able to identify NK cell activation in response to treatment directly—without the need for clustering, differential expression analyses, or additional technologies— highlighting the advantage of CoGAPS compared to standard analysis methods when studying tumoral immune cells. Using projectR to project the NK cell activation signature into several additional datasets allowed us to ultimately confirm that the transcriptional signature we identified in mice was clinically relevant in humans as well. This is particularly impressive when you factor in the known differences between mouse and human NK cell surface receptors and markers [76]. In this application, we use gene signatures from CoGAPS for projection and transfer learning. Other transfer learning methods have been developed to relate features in a target scRNA-seq dataset to a reference atlas, often relying on non-linear methods for feature identification [77, 78]. In contrast to these other approaches, our projectR software is robust for transfer learning from single-cell data (e.g., PCA, clustering, and other forms of linear matrix factorization) and may capture additional features of cell state transitions based upon all of these methodologies [7, 30]. Future extensions to projectR are needed to enable transfer learning from an ensemble of features across these latent space methods and from emerging non-linear methods for inference of more complex cell state transitions and gene regulatory networks.

The CoGAPS analysis of the scRNA-seq data from an immunotherapy-treated mouse model identified several immune cell states associated with treatment status, including the myeloid compartment. Notably, CoGAPS detected an M2 macrophage signature enriched in untreated mice and an M1 macrophage signature enriched in tumors from anti-PD-1 treated mice (Fig. 2D–G). We chose to focus our experimental validation on the NK cell activation signature identified by CoGAPS (pattern 7) for several reasons: (1) pattern 7 was the most clearly associated with a specific cell type and treatment, (2) increased expression of NK cell activation markers had been noted in anti-CTLA-4-treated mice from the original CyTOF analysis [4], (3) there is growing evidence that CTLA-4 is expressed by non-T cell human immune cell types [47–50], and (4) recent work found that human NK cells express PD-1 and are modulated by anti-PD-1 therapy [79, 80]. Therefore, we hypothesized that CTLA-4 was similarly expressed by human NK cells and activated by anti-CTLA-4 antibodies.

In addition to the experimental validation, our computational analysis with transfer learning demonstrated that the NK cell activation signature is associated with improved overall survival and anti-CTLA-4 response in melanoma patients. This signature was detected in anti-CTLA-4 responsive metastatic melanoma prior to the administration of treatment and correlated with response to therapy. This leads us to hypothesize that the presence of activated NK cells already within tumors improves tumor clearance mediated by anti-CTLA-4. The NK cell activation signature was also elevated in a patient that initially responded to a combination of anti-PD-1 and oncolytic virus therapy. This observation is consistent with previous studies showing that infection of tumors with oncolytic viruses can activate NK cells and stimulate NK-mediated anti-tumor immunity [81]. We note that this observation was specific to intratumoral NK cells and not present in circulating NK cells (Fig. 4E), indicating that approaches using peripheral blood to transcriptionally profile NK cell activation with respect to clinical outcomes may be limited. Future transfer learning analyses on large cohort studies of anti-CTLA-4-treated tumors with genomics data could further delineate the role of tumor NK cell activation as a potential predictive biomarker. However, these datasets are currently lacking in the literature, limiting our ability for such computational-driven biomarker analysis in this current study.

While our study is computationally focused, the application of our transfer learning pipeline for cross-species analysis to cancer immunotherapy still suggests that the role of NK cells in anti-CTLA-4 response is preserved between preclinical mouse models and human tumors. Despite growing evidence for the role of checkpoint receptors in NK cell-mediated anti-tumor responses, the expression of CTLA-4 in NK cells has been disputed in the literature for both mice and humans. Although mouse NK cells have been shown to inducibly express CTLA-4 in response to IL-2 [60], a recent study was unable to detect CTLA-4 on the surface of intratumoral mouse NK cells [42]. A study in humans also reported that NK cells from healthy donors do not express CTLA-4 [51]. Contrary to these earlier reports, our results demonstrate CTLA-4 is constitutively expressed by circulating healthy human donor NK cells and human NK cell lines. One possible explanation for why previous studies have failed to identify the expression of CTLA-4 by human NK cells is the reliance on flow cytometry in these studies. Flow cytometry can be limited by challenges related to the generation of antibodies and further complicated by the rapid surface expression dynamics of CTLA-4 [82]. In support of this explanation, we too fail to detect intracellular or surface CTLA-4 expression when using flow cytometry (Additional file 1: Fig. S3A and B), even though we are able to unequivocally demonstrate CTLA-4 expression at the RNA and protein level by qRT-PCR and western blot in ex vivo unstimulated healthy donor NK cells (Fig. 5B–E), as well as surface expression using immunofluorescence and biotinylation (Fig. 5G). Consistent with previous studies [83, 84], we show that human NK cells express CD28 and CD28H (Additional file 1: Fig. S4), a co-stimulatory receptor that competes with CTLA-4 for the binding of B7 ligands. The expression of B7 on tumor cells also enhances NK recognition and lysis of tumors through CD28-B7 interactions [83–89]. In addition, we show that CTLA-4 expression by human NK cells cultured in vitro is modulated in response to NK cell activation (Fig. 6). These findings suggest that CTLA-4 may have similar functions in NK cells and effector T cells [59]. Taken together, these results build upon previous studies that highlight a relationship between NK cells and anti-CTLA-4 response in humans. In melanoma patients treated with anti-CTLA-4, a higher percentage of circulating mature NK cells is correlated with improved overall survival, and NK cells isolated from responsive patients have increased cytolytic activity compared to NK cells isolated from non-responders [90]. In B16 melanoma models, NK cells and CD8+ T cells synergistically clear tumors in response to anti-CTLA-4 and IL-2 treatment [91]. Furthermore, anti-CTLA-4 has been shown to increase transcriptional markers of NK cell cytotoxic activity in CT26 colon carcinoma tumors [42]. While future mechanistic studies are needed to fully elucidate the specific function(s) of CTLA-4 in NK cell biology, these findings support the computationally driven translational approach employed in this study.

## Conclusions

As scRNA-seq datasets of immunotherapy-treated tumors become increasingly prevalent in cancer research, we need appropriate computational tools that can delineate actionable cellular mechanisms of action from these data. This inference can play a critical role in advancing basic science in the preclinical research pipeline, where relating findings to human datasets enables translation for precision immunotherapy strategies. This work describes a framework using latent space discovery through matrix factorization and transfer learning for cross-species data analysis which allows the integration of preclinical and clinical genomics datasets. We provide a powerful method for extrapolating relevant information while avoiding the unique biases of individual technologies (i.e., dropout in scRNA-seq, biased selection of genes in CyTOF, or aggregate transcriptional profiles in bulk RNA-seq). In addition, our approach enables the comparison of different tumor types and treatment conditions. While our study focused on the relation of preclinical models to human tumors, this approach can be readily applied within human tumors to relate mechanisms across tumor subtypes and can be broadly used in other disease contexts as well as drug repurposing. The ability to rapidly identify conserved therapeutic responses between mice and humans will help bridge basic science and clinical research to improve patient outcomes.

## Supplementary Information

**Additional file 1..** Contains all supplementary figures (Fig. S1 - S4).

**Additional file 2: Table S1**. CoGAPS 21 pattern matrix with pattern weights for each cell. **Table S2**. FDR adjusted gene set statistics for all 21 CoGAPS patterns. **Table S3**. Differentially expressed genes across pseudotime in NK cells collected from tumors treated with anti-CTLA-4. **Table S4**. Correlation values and p-values for each of the 21 CoGAPS patterns and the CIBERSORT NK cell activation signature. **Table S5**. Correlation values and p-values for CIBERSORT cell type estimation and CTLA-4 expression in tumors from TCGA.

## Data Availability

The scRNA-seq datasets analyzed during the current study are available in NCBI’s Gene Expression Omnibus (GEO). Mouse scRNA-seq data from Gubin et al. [4] was downloaded from GEO (accession number GSE119352) at https://www.ncbi.nlm.nih.gov/geo/query/acc.cgi?acc=GSE119352. Paired mass cytometry data from Gubin et al. [4] was downloaded from the FLOW Repository (FR-FCM-ZYPM) at http://flowrepository.org/id/FR-FCM-ZYPM. Human metastatic melanoma scRNA-seq data from Sade-Feldman et al. [5] and de Andrade et al. [6] was downloaded from GEO (accession number GSE120575) at https://www.ncbi.nlm.nih.gov/geo/query/acc.cgi?acc=GSE120575 and (accession number: GSE139249) at https://www.ncbi.nlm.nih.gov/geo/query/acc.cgi?acc=GSE139249 respectively. Bulk RNA-seq data was downloaded from The Cancer Genome Atlas [18]. Code used for analysis in this manuscript is available on Github at https://github.com/edavis71/projectR_ICI [92].
