# Supplementary material for: Transfer learning between preclinical models and human tumors identifies a conserved NK cell activation signature in anti-CTLA-4 responsive tumors
Source: Genome Med. 2021 Aug 11;13:129. doi: 10.1186/s13073-021-00944-5 (PMC8356429; doi:10.1186/s13073-021-00944-5)
Supplement: Supplementary file 1 — Additional file 1.. Contains all supplementary figures (Fig. S1 - S4). [file 13073_2021_944_MOESM1_ESM.pdf]

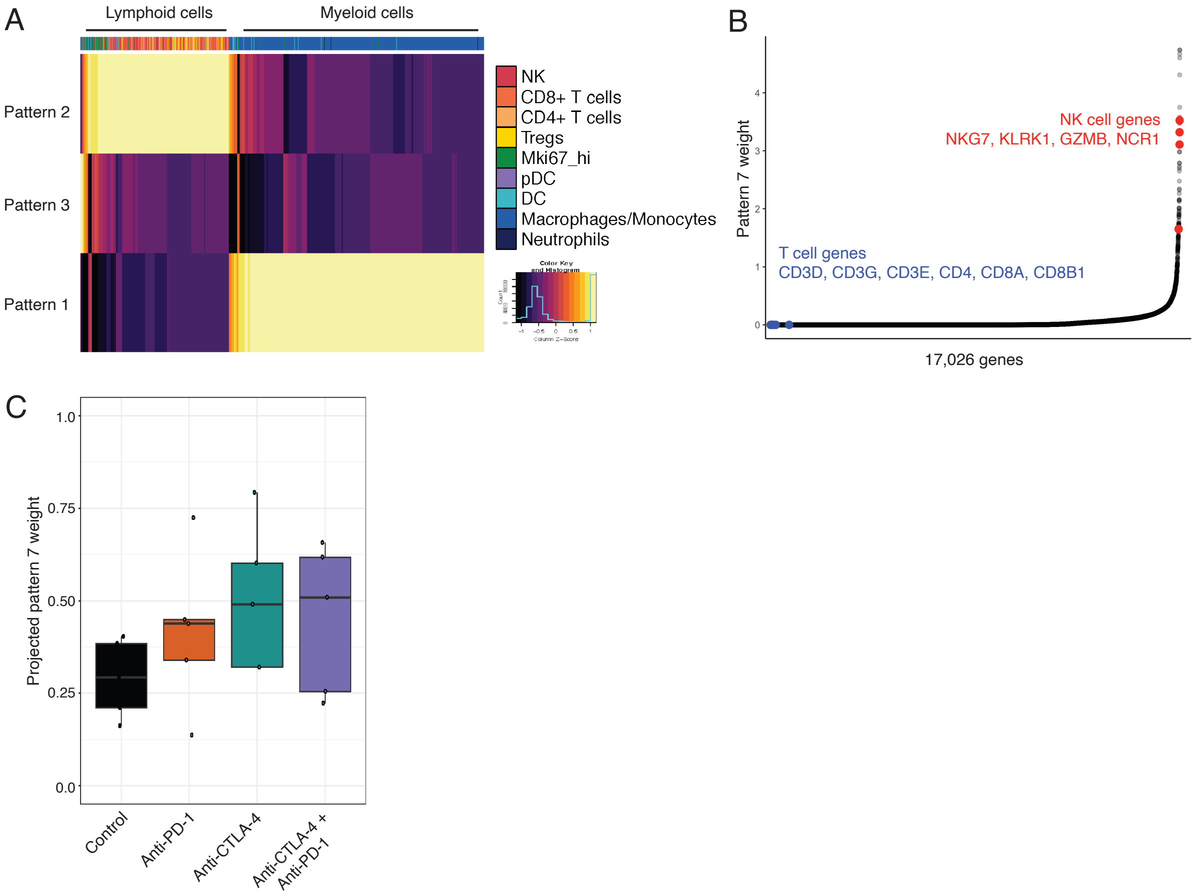

Figure S1: CoGAPS patterns identify immune cell lineage and transfer across data modalities. A. Heatmap of transcriptional signatures (patterns) identified with CoGAPS. When CoGAPS is performed at low dimensionality, here being 3 patterns, the identified transcriptional signatures segregate cells by immune cell lineage. Pattern 3 is relatively flat across all cells, while patterns 1 and 2 define myeloid and lymphoid lineage cells, respectively. **B.** Scatter plot of pattern 7 gene (amplitude) weights for all 17,026 genes included in the CoGAPS result object, indicating that pattern 7 is specific for NK cell marker genes compared to T cell marker genes. **C.** Boxplot of the projected NK cell activation signature (pattern 7) weights in tumor infiltrates from mouse tumors analyzed by mass cytometry on day 11 after treatment. Each point represents a replicate sample. For each replicate, the mean protein expression of 37 genes was used as input for projectR. The NK cell activation signature is highest in lymphocyte samples treated with anti-CTLA-4, either alone or in combination with anti-PD-1.

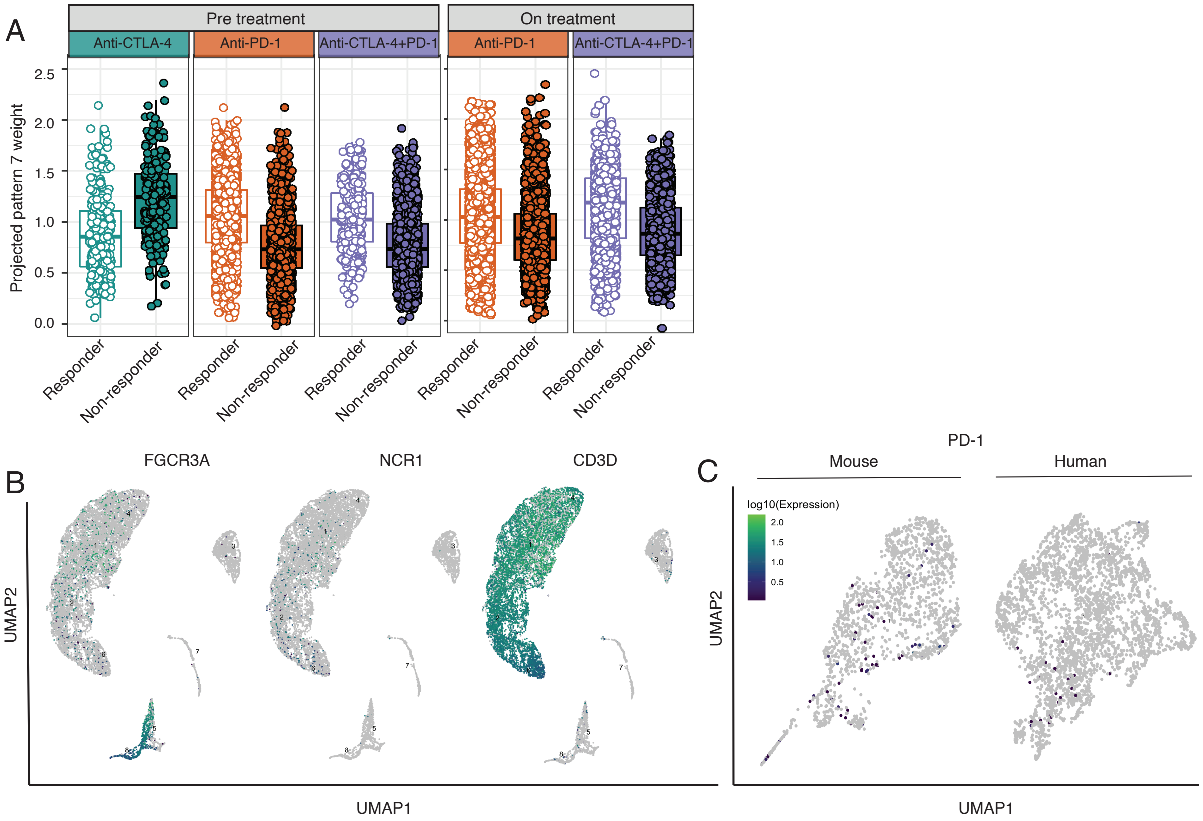

Figure S2: NK cell activation signature is associated with anti-CTLA-4 response. A. Box plot of projected pattern 7 weights across intratumoral immune cells from metastatic melanoma patients prior to ICI treatment [5]**.** Cells are colored by therapy and separated by patient response. Increased pattern 7 is associated with immune cells from patients responsive to anti-CTLA-4. **B.** UMAP dimension reduction with cells colored by single-cell gene expression for representative NK and T cell marker genes. **C.** UMAP dimension reduction with cells colored by single-cell gene expression for PD-1 in mouse (left) and human (right) intratumoral NK cells. Activated NK cells are known to express PD-1, demonstrating that the observed pattern of PD-1 expression is consistent with the reduced ability of scRNA-seq to capture low to moderate expressed genes.

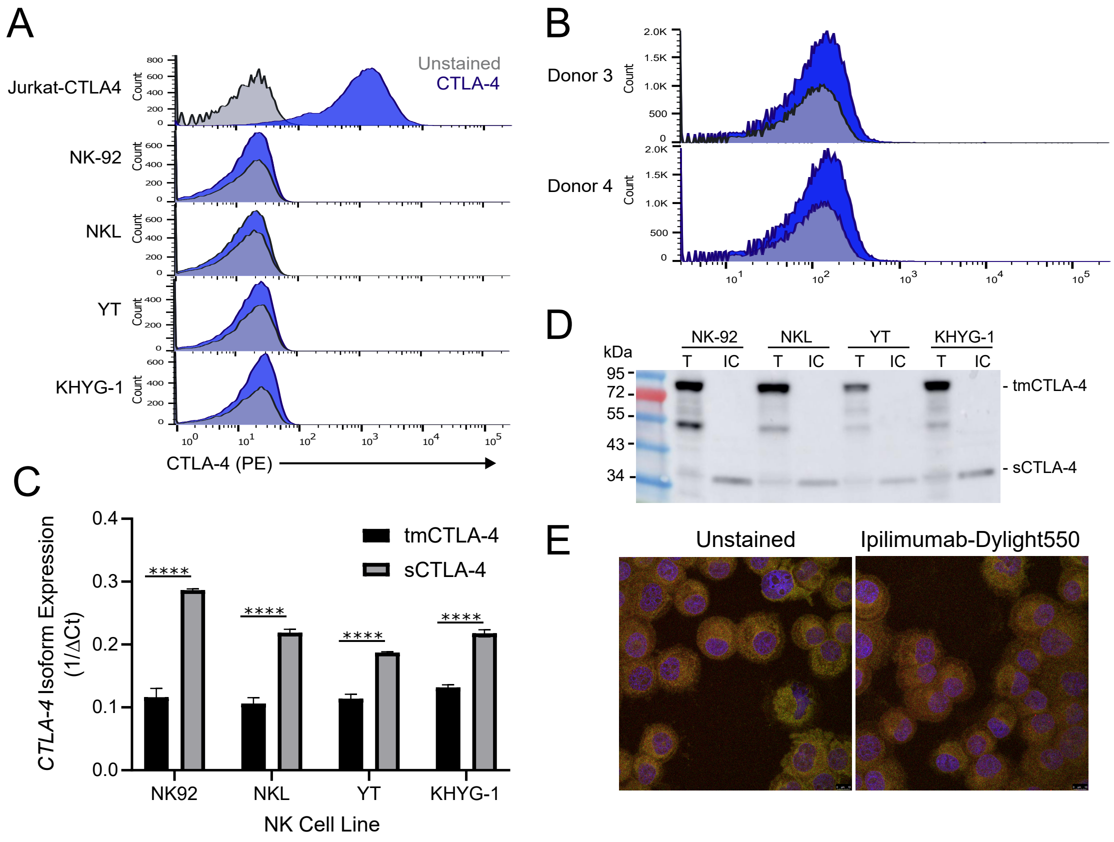

Figure S3. Human NK cells express CTLA-4. A. Flow cytometry for surface expression of CTLA-4 in positive control (Jurkat-CTLA4) and NK cell lines (NK-92, NKL, YT, KHYG-1). **B.** Flow cytometry for surface expression of CTLA-4 on CD56+ selected *ex vivo* unstimulated NK cells derived from healthy human donors **C.** Quantitative qRT-PCR analysis of transmembrane (tmCTLA-4) and soluble (sCTLA-4) isoforms in human NK cell lines, (n=6, p-value < 0.001 = ****). **D.** Western blot of total protein (T) and intracellular (IC) protein isolated from human NK cell lines NK-92, NKL, YT and KHYG-1 using cell surface protein biotinylation for exclusion of surface proteins demonstrating surface expression of CTLA-4 dimers and intracellular expression of CTLA-4 monomers. Western blot is representative of two independent experiments. **E.** Immunofluorescent images of PANC-1 cells stained with Dylight550-labelled ipilimumab. Blue staining indicates DAPI. Shown are representative images of a single field of view taken via confocal microscopy (magnification, 63X).

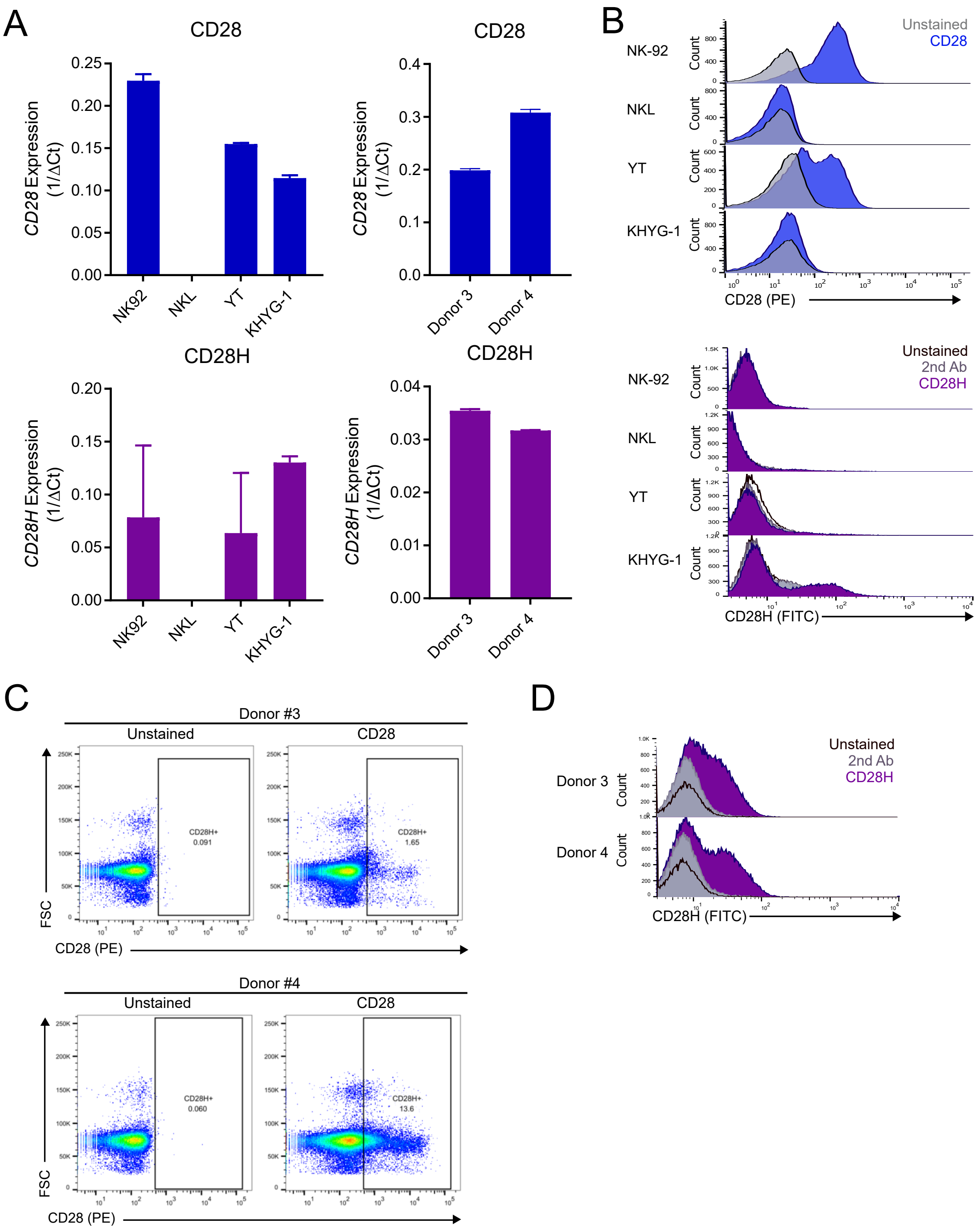

Figure S4. CD28 and CD28H expression on human NK cells. A. qRT-PCR assessment of CD28 and CD28H expression in human NK cell lines and primary donor NK cells, (n=6 for NK cell lines, n= 2 for donor NK cells). **B.** Flow cytometry assessment of CD28 and CD28H surface expression by human NK cell lines **C.** Flow cytometry assessment of CD28 surface expression by primary donor NK cells. **D.** Flow cytometry assessment of CD28H surface expression by primary donor NK cells.

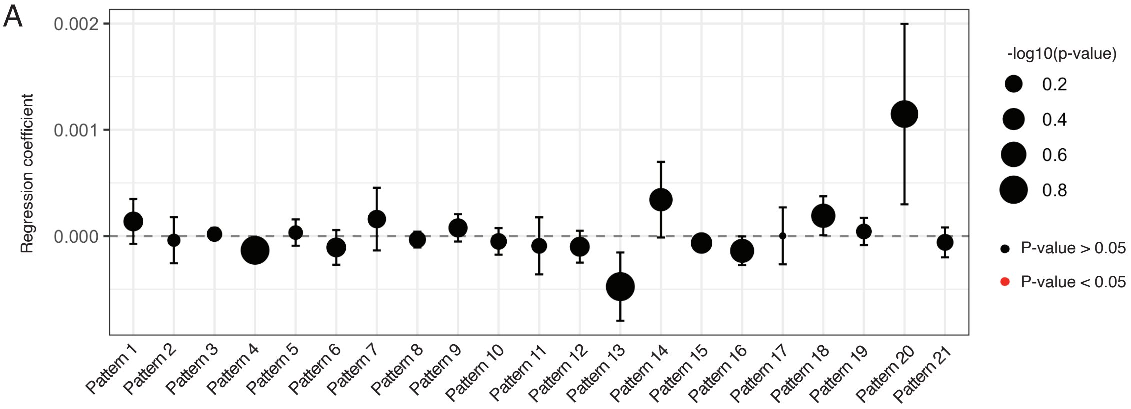

Figure S5: Regression coefficients of pattern associations with TCGA tumor survival. A. Regression coefficients of an age-adjusted multivariate Cox proportional hazards regression model that relates CoGAPS patterns and overall survival in primary melanoma lesions from TCGA. Point size scaled to the coefficient's p-value. Red points indicate patterns with significant coefficients. No regression coefficients for any CoGAPS pattern are significant in primary melanoma.
